# Extra-Uterine Pregnancy; Death and Removal of Fœtus by Prof. J. P. White, M. D.

**Published:** 1880-03

**Authors:** William D. Granger


### Clinical Reports.

### EXTRA-UTERINE PREGNANCY; DEATH AND RE-REMOVAL OF FŒTUS—BY PROF. JAMES P. WHITE, M. D.

REPORTED BY WM. D. GRANGER, M. D.

THE following record, made by Dr. White, when he first saw the patient, will best serve our purpose in presenting the early history of this case.

"Mrs. G., age 26; menstruated at 15, married at 19; first pregnancy; pregnancy suspected December, 1878; menstruation, or irregular losses of blood continued during the succeeding three or four months; slight, but frequent; saw no shreds or membranes in the discharge; after three or four months flow less frequent; felt life at the expected time, fore part of April, 1879; suffered a great deal of pain in the lower part of abdomen, and the child's motions gave greatly increased pain; motions continued frequent, and were plainly perceptible to husband up to the following July, when they suddenly ceased; the abdomen diminished in size, the breast became flaccid; and all symptoms of pregnancy, except abdominal enlargement, disappeared; menstruated in August and September, but has not been regular since. About November 1st, Dr. Varian, her physician, dilated the os with sponge tents, so as to enable him, as he told her, to pass his finger to the fundus; found nothing there. Dr. V. diagnosticated extra-uterine pregnancy. Nov. 11, 1879, patient came to me and I made a careful examination. Tumor on right side, and nearly up to unbilicus; tumor quite solid; think I can make out the lower extremities of fœtus; abdomen tender, and has been since the dilatation of the os; tumor extends downward in the vaginal cul-de-sac; os patulous; sound enters into the organ 3½ inches; nothing to feel in the cavity by sound; general health quite good; confirmed diagnosis of extra-uterine pregnancy, with death of fœtus; recommended tonic and good diet, and waiting for indications." Dr. Varian, of Titusville, Pa., in his notes says, that in March, 1879, patient was supposed to be threatened with miscarriage; that the pains simulating labor pains, had been of daily occurrence; vomiting was violent, frequent and stercoraceous, and she was greatly emaciated; under treatment patient regained fair health. Again in May, patient had persistent vomiting, and again in August, but the uterine pains and the hemorrhage did not return. About two weeks after, Dr. White saw the patient; she suffered from prolonged vomiting, and also had abdominal pains, though not very severe. At Dr. White's request, the reporter wrote the husband, advising in case the stomach refused to perform its work to sustain her by rectal alimentation, and, if in spite of this, patient lost in weight, strength and health, or if local inflammation seemed to be ensuing, or if the tumor seemed to be pointing, advised an operation for the removal of child, and so assist nature in the performance of a work, because the effort to do it, if unaided, would probably destroy the life of the mother. The patient was reported as improved. But this soon proved apparent, not real, for a month later, in January, she was reported to be losing in health and flesh, and unable to retain food. The advice for an operation was again unheeded. Early in February she was reported much worse, and an operation, delayed two months against advice, consented to. The operation was performed February 7th, in the presence of a large number of physicians. Dr. White was assisted by Dr. Varian, Dr. Thomas Lothrop, Dr. O'Brien, and the writer. Dr. Barr, of Titusville, administered the ether. Before beginning the operation, Dr. White stated at length the history and diagnosis of the case, substantially as given in this article. He would operate because the patient's life depended upon it; she was emaciated to the last degree; was very anæmic; she could retain no food in the stomach, and she could live but a few days longer. With such a decaying mass and accumulation of pus in the body, the blood was poisoned, causing the vomiting, interfering with assimilation of food introduced by rectum, and therefore bringing on the anæmia, loss of flesh, and the great prostration from which she is now suffering. As for the operation, the object is to remove the child; whether the placenta would be removed, would depend upon its situation and its attachments; he would prefer to remove it if it could be easily accomplished. Exactly *how* the operation would be performed, it was impossible to fore-tell. It was one of expectation; each step would have to be deliberately decided upon before taken. He intended, if possible, to make an exploratory incision upon the median line, between the umbilicus and pubis, expose the sac, stitch it to the parietes to prevent overflow of fluids into the peritoneal cavity, and enlarge the opening sufficient to extract the fœtus. When examined previous to the operation, there was no projecting tumor in the abdomen to be seen, only a little fullness; but a hard tumor with distinct outlines was easily felt. The exploratory incision, two inches long, was made, the parietes carefully dissected and the sac exposed; the opening was enlarged to about four inches; the tumor being more or less adherent to the walls of the abdomen, a small part of the upper end of the wound, which extended slightly beyond the sac, was the only opening directly into the peritoneum. The degenerated and friable sac easily ruptured when examined, and foul and offensive gas and fluid escaped; the opening in the sac was quickly enlarged, and a well-developed female fœtus of about six and one-half months was extracted by its feet. The head was entirely destroyed, and the occipital and two parietal bones were afterwards taken out from the sac. With the exception that the floating ribs had perforated the skin, there was but little other signs of advanced decay. The placenta was not found attached, but broken-down portions of it were found in the sac, and together with a thick fluid, partly pus and partly broken-down tissue, was removed, and the sac thoroughly cleansed by sponges, washed in strong carbolic acid water. The sac extended into the pelvic cavity, and a finger introduced into the sac, met in Douglas' cul-de-sac, with only a thin wall dividing them, a finger introduced into the vagina. A trocar was passed from the most dependent part of the sac into the vagina, and a medium-sized, gum-elastic catheter was introduced, tied with a ligature, which came through the abdominal wound, and was left in place to serve as a drainage tube. The wound was brought together by deep silver stitches, which included the sac, and by superficial stitches between. Very little blood was lost during the operation. It is impossible to say just what variety of uterine pregnancy this case presented. If it had been tubal, it most probably would have ruptured during the severe pains of the first few months. Beside the uterus and the right ovi-duct were in position. The uterus could be easily felt before the operation in front of the tumor. It probably was some of the forms of abdominal pregnancy. The ovarian form while it is denied by some writers, is certainly very rarely seen and did not give evidence of being present in this case.

For the first week after the operation, the patient was under my care. She rallied well from the shock of the operation, and that evening had a good pulse of 128, and a temperature of 99½. The highest temperature was on the evening of the fourth day, 100¼; on the evening of the seventh day the pulse was 110; temperature, 99½; the next morning, pulse 112; temperature, 97½; patient improved steadily after the operation; she vomited the little food given until the seventh day; the stomach was given a perfect rest, and food was introduced by the rectum only; whiskey, beef tea, milk and egg were freely given; also, quinine, gr. v., dialysed iron, mm. xx three times a day. At the end of the week patient had lost the pinched starved look; her lips were red; she had gained greatly in strength and a little in flesh. On the seventh day she began to take small quantities of food by the stomach, which was well borne. The discharge from the sac was profuse and very offensive, but diminished and became less offensive at the end of the week. The sac was syringed out twice a day with a five per cent. solution of carbolic water, several quarts being used, until it ran from the drainage tube a clear stream.

After Saturday, Feb. 14th, the case came under the charge of the Drs. Waid, of Spartansburgh, Pa. They report improvement up to Thursday, (the 12th day after the operation); the patient taking soft boiled eggs, milk and beef tea by the mouth; the silver stitches were taken out, and the discharge became much less, and all except a point at the lower edge of wound, and near the upper edge, where discharges continued to come, was granulating. Thursday the doctors began to suspect that undigested food was coming from the openings, but not until Saturday, Feb. 21st, when it became abundant, could they clearly make out undigested milk, egg, etc.; the patient's condition changed, and she began to lose strength, and to have a quickened and weakened pulse, though at no time did the thermometer show any fever; at the time the discharge of food began, the patient's appetite enormously increased, and continued until the second operation. A consultation was held on Monday, Feb. 23d, Dr. White unfortunately not being able to be present, and it was decided to re-open the abdominal wound, find out the point where the intestine was ruptured, and if possible close the opening. The operation was performed by Dr. Varian, assisted by the Drs. Waid and the reporter; the external abdominal wound was easily separated, and the remains of the sac exposed; the sac was mostly obliterated, its opposite walls being in apposition and bound together by adhesions of newly formed tissue; projecting into the sac through the right wall, about an inch below the surface, and about midway between the upper and lower extremity of the external wound, was found a bone, which, being withdrawn proved to be one-half the lower jaw of the fœtus; in this sac were other small pieces of bone, tissue with hair upon it, and food; at the upper right-hand corner of the sac was found an opening into a large intestine; the outer wall of this sac, through the integuments, was opened to the extent of about three inches, the incision being at right angles with the original wound; the epigrastric artery was cut, and both ends ligated; the opening into the intestine was large enough to admit two fingers; it appeared at first as though the gut was cut across and ended in the sac, but by passing two fingers into the gut, in different directions, it was found that there was a wall between them; the wall of the sac, and the adhesions of the intestine to it were broken down, the intestines drawn out, when a longitudinal rent, two and one-fourth inches long, with ragged edges, was found; about the original seat of the ulceration the tissue was a good deal degenerated; the edges of the wound were turned in, the peritoneal surfaces brought together and stitched with carbolized cat gut, the narrow cul-de-sac extending downward into the pelvis, spoken of the first operation, was not wholly healed, although partially bridged over, and in it was found broken down tissue, small pieces of bone, etc.; it was cleansed, and a drainage tube inserted; the external wound was brought together by silver ligatures; the patient did not rally well, although she showed considerable strength a few hours after the operation, and died of shock and exhaustion six hours after being placed in her bed. It is safe to say, that had not this most unfortunate complication ensued, the patient would have recovered, for her rapid gain until this accident, and the almost entire obliteration of the large sac, warranted the first operation, which fairly may be said to be successful. Dr. White, Dr. Varian, and others, at the first operation, very carefully examined the sac before it was closed, and found nothing to cause them to think that any such complication as this was likely to occur; this bone and other tissue had evidently been completely shut off from parent sac, and had formed, before the first operation, an entirely separate one of its own; the great destruction and weakening of its walls, as shown by the profuse discharge and the contraction of the sac, so that its opposite walls had come together, on the one hand, and the firm adhesions of the intestines to the sac on the other had allowed the ends of the encysted bone to penetrate both the cavity of the parent sac and the intestine; the great rent in the intestine was caused, I think, by a weakening of its walls by inflammatory processes, for it had a red, turgid, "angry" look at the time of the last operation, and by the shrinking of the large sac, and the consequent pulling of the adherent intestine from its natural position, which had first forcibly torn the walls of the ulcer, and the contraction of the sac and counter-pulling of the intestines continuing, the large fissure was next made. It is safe to say, that had the operation been done when first advised in December, the patient would be alive to-day.

